# Mechanisms of Hydroxyl Radical Chemistry in Aqueous Solution Triggered by Photoexcitation and Probed by Soft X‑rays

**DOI:** 10.1021/jacs.5c20053

**Published:** 2026-02-17

**Authors:** Leo Cordsmeier, Wagner Ribeiro da Silva Neto, Mattis Fondell, Rolf Mitzner, Vinícius Vaz da Cruz, Sebastian Eckert, Alexander Föhlisch

**Affiliations:** † Institut für Physik und Astronomie, Universität Potsdam, Karl-Liebknecht-Straße 24/25, 14476 Potsdam, Germany; ‡ Institute for Methods and Instrumentation for Synchrotron Radiation Research, 545117 Helmholtz-Zentrum Berlin für Materialien und Energie GmbH, Hahn-Meitner-Platz 1, 14109 Berlin, Germany

## Abstract

Hydroxyl radicals are among the most important radicals on earth, being present in the human body, the atmosphere, rivers, and oceans, contributing to mechanisms like oxidative stress in cells and the photochemistry of the troposphere, and posing a threat to aquatic life. Extensive use of fertilizers in agriculture has led to increased levels of nitrogen oxides in many rivers around the world, which are a major source of hydroxyl radicals in water. In this paper, we explore the photoinduced generation of hydroxyl radicals from nitrite and their scavenging by the radical scavenger 2,2,6,6-tetramethylpiperidinyloxyl (TEMPO) in aqueous solutions using transient soft X-ray absorption spectroscopy (XAS) at the oxygen and nitrogen K-edges. We show the photoinduced generation of hydroxyl radicals from nitrite and determine its mechanism. For the scavenging of hydroxyl radicals by TEMPO, we show that the mechanism does not proceed through a bound intermediate state between the two molecules, as has been proposed in the literature, but instead through an electron transfer.

## Graphical abstract

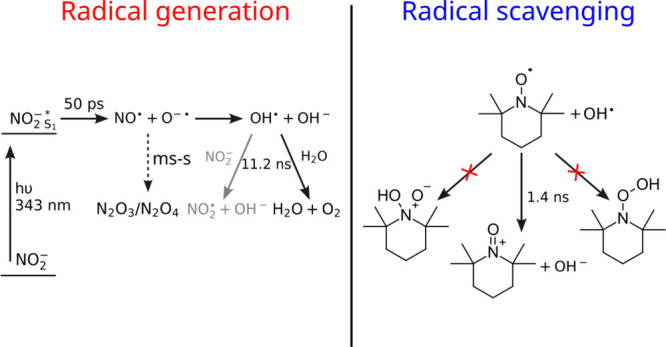

## Introduction

Reactive oxygen species (ROS) play an important role in many areas, ranging from atmospheric chemistry to wastewater treatment and in the human body.^1–3^ Among them, OH^·^ radicals are especially noteworthy due to their prevalence and high reactivity.^4^ They can be found in the photochemistry of the troposphere and in cells in the human body, causing oxidative stress, and have been linked to skin cancer and the aging process.^5,6^ They are also becoming more common in waterways like rivers and are used in water treatment plants to oxidize organic material.^7^

Hydroxyl radicals (OH^·^) were first generated and measured in 1946 using Fenton's reaction,^8^ but can also be generated using H_2_O_2_, UV irradiation of water, or by nitrogen oxides.^9^ This last group is becoming increasingly relevant in recent years due to the extensive use of nitrogen-containing fertilizers in agriculture, which leach into the groundwater. The photolysis of nitrite (NO_2_^–^) and nitrate (NO_3_^–^) generates OH^·^ in aqueous solution, among a range of other radicals, leading to a rich photochemistry of both species in aqueous environments.^10^ Because of their high reactivity, the detection and quantification of these radicals have presented a significant challenge. Methods for the detection of radicals generated in solution include laser flash photolysis^11^ and electron paramagnetic resonance (EPR)^12,13^ and the detection and analysis of reaction products of these radicals with other molecules, such as radical scavengers, which form stable and long-lived products that can be detected using traditional spectroscopic methods.^14^

One class of radical scavengers is nitrones such as 2,2,6,6-tetramethylpiperidinyloxyl (TEMPO). These compounds are stable molecules despite being radicals themselves, and are excellent spin traps commonly used in EPR,^15^ as well as efficient radical scavengers. Their stability arises from the delocalization of the unpaired electron between the nitrogen and oxygen, and from bulky methyl groups shielding the radical center sterically.^16^ These combined factors make TEMPO stable at room temperature both as a solid and in solution. This stability has made it useful for other applications beyond radical scavenging, like nitroxide-mediated polymerization,^17,18^ as a catalyst in organic synthesis,^19–21^ or as a cathode material in redox flow batteries.^22,23^

In general, the deactivation of OH^·^ can occur through three main pathways. First is the recombination to form H_2_O_2_ or the combination with other radicals to form stable products. The second pathway is hydrogen abstraction from another molecule acting as a scavenger, such as MeOH, to form H_2_O and a new radical, MeO^·^. The last pathway is electron transfer from another molecule to form OH^–^. While these three pathways are known and can be investigated by analyzing recombination and scavenging products, the direct observation of the respective intermediates remains challenging due to the high reactivity of OH^·^ and other radicals.

In this study, picosecond soft X-ray spectroscopy at the nitrogen and oxygen K-edges is used in combination with an optical laser as a pump to determine the mechanisms of the photoinduced generation of OH^·^ from nitrite and the scavenging mechanisms of OH^·^ using nitroxyl radicals, specifically TEMPO (Figure 1). X-ray absorption spectroscopy at the oxygen and nitrogen K-edges probes the unoccupied 2p valence density of states at the oxygen and nitrogen sites in an element-specific way with chemical state selectivity. In our experiment, all involved species contain nitrogen and/or oxygen moieties that are thus accessible. In particular, the electronic structure changes in NO_2_^–^, whose ground-state electronic structure has recently been investigated using X-ray absorption spectroscopy and resonant inelastic X-ray scattering,^24^ and those of TEMPO can be monitored based on its absorption signatures at the nitrogen K-edge. These features are fully separated from those of OH^·^, which is monitored on the oxygen K-edge. The dynamic and kinetic information is then gained by time-resolved laser pump-X-ray probe with a time resolution of 80 ps,^25,26^ tracing the evolution of the radical chemistry. With this approach, we investigate the generation of OH^·^, its lifetime under different scavenging conditions, and the relevant intermediate species in its scavenging and generation processes in situ.

**Figure 1:**
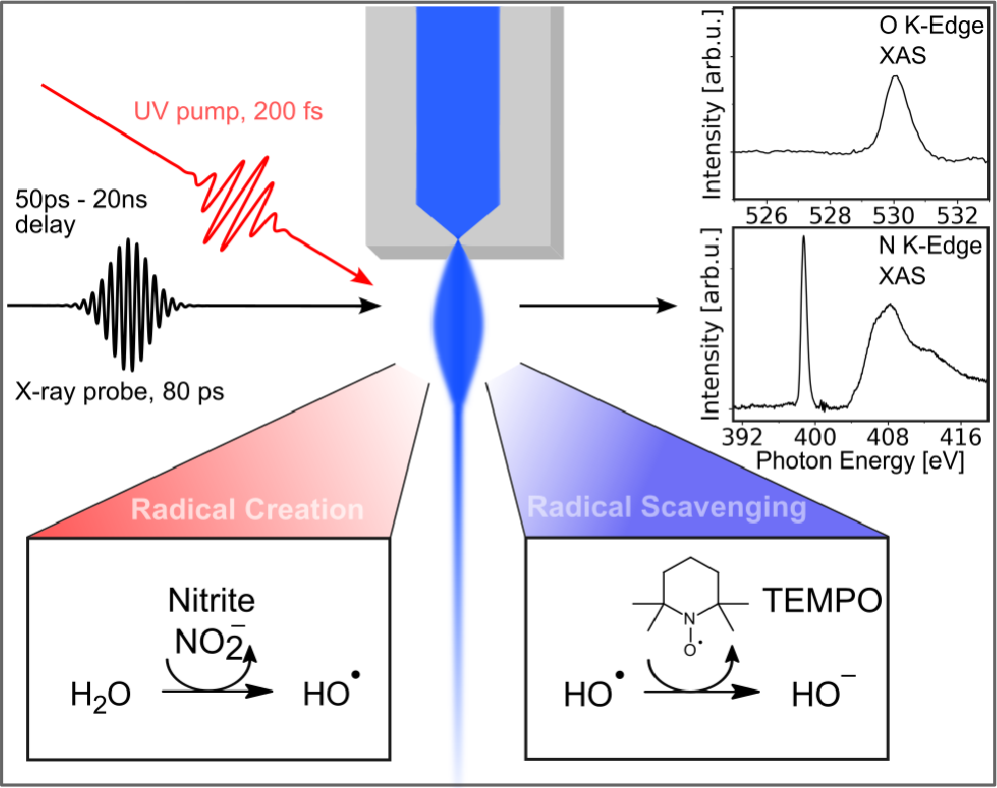
Temporal evolution of radical creation and scavenging from optically excited soft-state X-ray absorption spectroscopy in aqueous solution. The unoccupied 2p-valence density of states at nitrogen and oxygen sites is traced at the nitrogen and oxygen K-edges, respectively, in an element-specific and chemical state-selective way. The aqueous solution sample is delivered by a liquid flat-jet from a microfluidic chip nozzle. The X-rays are transmitted normally through the liquid sheet to be detected by a photodiode behind the flat-jet. Femtosecond UV laser pulses with variable wavelength are used for optical pumping.

## Results and Discussion

Let us begin by investigating the photoinduced generation of OH^·^ from NO_2_^–^, the mechanism of which has been proposed by Mack et al.^9^ To briefly summarize the initial steps, which are most relevant on short time scales, after an initial excitation into the S_1_ state of NO_2_^–^ the molecule dissociates into NO^·^ and O^·^^–^, the latter rapidly hydrolyzing with the surrounding water to OH^·^. It is proposed that OH^·^ is then efficiently scavenged by NO_2_^–^ to form NO_2_^·^ and a range of other nitrogen–oxygen compounds.

The UV–vis spectrum of NO_2_^–^ (inset in Figure 2a) shows an optically weak HOMO → LUMO transition at 360 nm, with the wavelength of the laser used in this experiment being marked at 343 nm. In Figure 2b, the transient oxygen K-edge X-ray absorption spectra after optical excitation at 343 nm of NO_2_^–^ at different delays, as well as a reference spectrum of H_2_O, are shown. The formation of OH^·^ (526 eV, magenta line) can be observed after laser excitation at 343 nm in the NO_2_^–^ spectrum but not in the reference water spectrum. In both of these experiments, a laser fluence of 60 mJ/cm^2^ has been used. This indicates that the formation of OH^·^ is not a result of radiation-induced water cleavage, but a product of the photochemistry of NO_2_^–^. Further, the bleaching of the π* resonance of NO_2_^–^ (531.65 eV, red line) and the π* resonance of NO^·^ (533.15 eV, blue line) are in good agreement with our calculations (Figure 2c), which were performed using the ORCA package,^27,28^ and the static spectrum of NO_2_^–^ in Figure 2a. The feature at 528.4 eV (green line) is attributed to the first excited state (NO_2_^–*^) of NO_2_^–^ after excitation at 343 nm. In the time traces in Figure 2g, the excited state is formed right after excitation from the ground state and dissociates within 100 ps into NO^·^ and O^·^^–^, which rapidly hydrolyzes the surrounding water to form OH^·^. The NO^·^ formed does not react further on the time scales investigated in this experiment, which agrees with the literature stating its lifetime to be in the millisecond-to-seconds range.^29,30^

**Figure 2:**
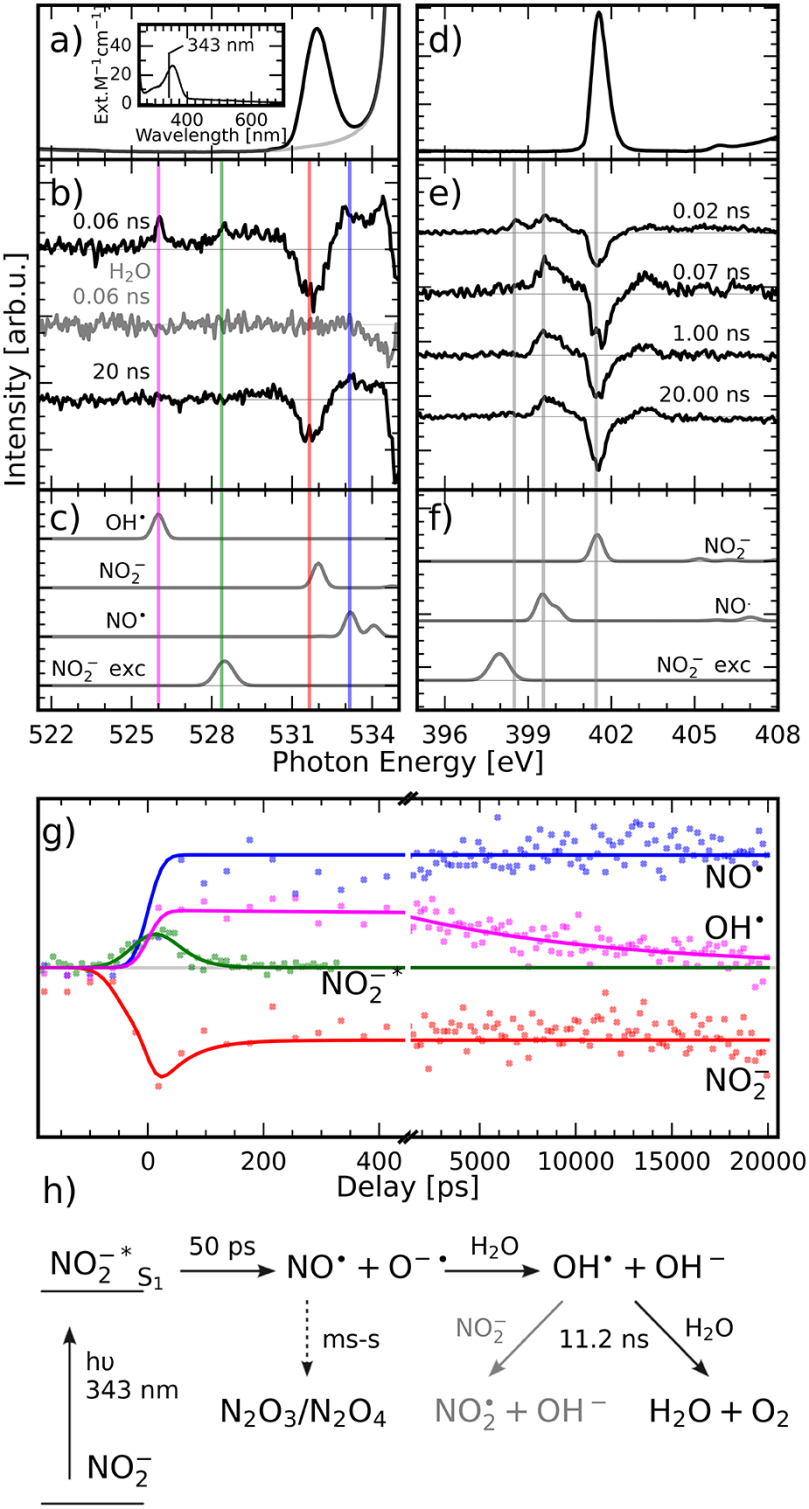
Dynamic evolution of NO_2_^–^ in aqueous solution from time-resolved X-ray absorption after photoexcitation at 343 nm. (a) Static oxygen K-edge spectrum of NO_2_^–^ in water (black) and pure water (gray), the inset shows the UV–vis spectrum of NO_2_^–^ with the excitation energy marked at 343 nm. (b) Oxygen K-edge pump probe spectra of NO_2_^–^ and H_2_O taken at different delays after excitation at 343 nm. (c) Calculations of O 1s → HOMO transition for different species using PBE0/TD-DFT. (d) Static nitrogen K-edge spectrum of the ground state. (e) Nitrogen K-edge pump probe spectra of NO_2_^–^ taken at different delays after excitation at 343 nm. (f) Calculations of N 1s → HOMO transition for different species using PBE0/TD-DFT. The spectra are offset vertically for visibility. (g) Time traces from 0.2 ns before excitation at 343 nm until 20 ns after excitation are shown. The selected energies correspond to the energies marked in (b) and (c) by vertical lines; for details, see Discussion. (h) Summary of decay pathways and their time scales involved in the photoinduced decay of NO_2_^–^. The static and transient X-ray absorption spectra were taken using 500 mM NO_2_^–^ in water.

Finally, the time trace of the OH^·^ (526 eV, magenta) generated from the photodissociation of NO_2_^–^ completely decays over 20 ns with τ = 11.1 ± 0.5 ns. This is unexpected, because OH^·^ is a key species in the regeneration of NO_2_^–^ after photolysis.^9^ However, this process is claimed to occur on the millisecond-to-second time scale, long after we observed the complete decay of OH^·^ in our experiment. While the regeneration of NO_2_^–^ cannot be observed in this study, it can be assumed that the regeneration of NO_2_^–^ occurs through the recombination of NO_*x*_ radicals and their reactions with water and not by reaction with OH^·^. To further investigate the photoinduced generation of OH^·^, another experiment using H_2_O_2_ was conducted (Figures S1 and S2) to determine the lifetime of OH^·^ in the absence of any potential radical scavenger as well as with increasing concentrations of MeOH. From this experiment, a lifetime of OH^·^ of τ = 10.1 ± 0.3 ns in the absence of any scavengers and a minimum lifetime of τ = 1.3 ± 0.1 ns in the presence of 1 M MeOH, at which point additional MeOH has no more effect on the lifetime of OH^·^, were determined. Based on these results, it can be assumed that the main deactivation pathway of OH^·^ in this reaction is through recombination and interactions with water,^31^ and that the generation of NO_2_^·^ and other scavenging products of OH^·^ by NO_2_^–^ is negligible, as the lifetime of OH^·^ is unaffected by the presence of NO_2_^–^. In Figure 2d, the Nitrogen K-edge X-ray absorption spectrum of the ground state of NO_2_^–^ is shown with the feature at 401.45 eV attributed to the N 1s → π* transition. In the transient absorption spectra (Figure 2e), three features can be attributed to the bleaching of the π* resonance of NO_2_^–^ (marked at 401.45 eV),^32^ the excited state NO_2_^–*^ (marked at 398.5 eV), and finally the π* resonance of NO^·^ ^33^ (marked at 399.55 eV). Time traces at the three energies indicated in Figure 2e are shown in Figure S4. There, it can be seen that the signal attributed to NO_2_^–*^ has a lifetime τ = 29 ± 19 ps and is only visible in the initial scan at a 0.02 ns delay in Figure 2e, while a complementary feature is found below 200 ps in the ground-state trace (red) caused by the relaxation of the excited state. These features agree with the lifetime determined from the time traces on the oxygen K-edge (Figure 2g), while the other two signals do not change over the 20 ns investigated in this study.

We propose the mechanism of the formation to be according to Figure 2h. After an initial HOMO → LUMO excitation of NO_2_^–^ into the first excited state, the molecule dissociates into NO^·^ and O^·^^–^. O^·^^–^ quickly hydrolyzes to form OH^·^. On longer time scales, NO^·^ might further react with OH^·^ to form NO_2_^·^ and a range of other products, which could not be observed in this experiment.^9^ Additionally, we could not observe any reaction of OH^·^ with NO_2_^–^ before the radicals reacted with the surrounding water and recombined.

Let us now focus on the radical scavenging of OH^·^ by TEMPO and briefly discuss the electronic structure of TEMPO and its redox forms, involved in the scavenging process (Figure 3). The nitrogen K-edge absorption spectrum of TEMPO is characterized by two features. The intense signal at 398.6 eV in Figure 3b corresponds to the N 1s → π* excitation into the SOMO of TEMPO, while the continuum of σ* transitions lies between 405 and 415 eV. The nitrogen K-edge spectrum of TEMPO^+^ shows the same features shifted to higher energies by approximately 1.5 eV. This is explained by the reduced screening of the core potential by the positive charge in TEMPO^+^ compared to TEMPO, resulting in lower core level energies. For TEMPOH, the opposite effect can be observed; the additional electron fills the π* orbital, resulting in stronger core-level screening, while the N 1s → π* transition disappears, because the orbital is now filled with two electrons. The impact of the valence electron structure, by protonation or electron transfer, on the core-level binding energies is a common signature in K-edge soft X-ray absorption spectroscopy.^34–37^ In the oxygen K-edge spectra, only the O 1s → π* transition can be observed, because the σ* transitions lie above 535 eV and therefore overlap with the pre-edge region of the water spectrum. At the oxygen K-edge, the spectrum of TEMPO^+^ is shifted compared to the TEMPO spectrum, like in the nitrogen K-edge spectra.

**Figure 3:**
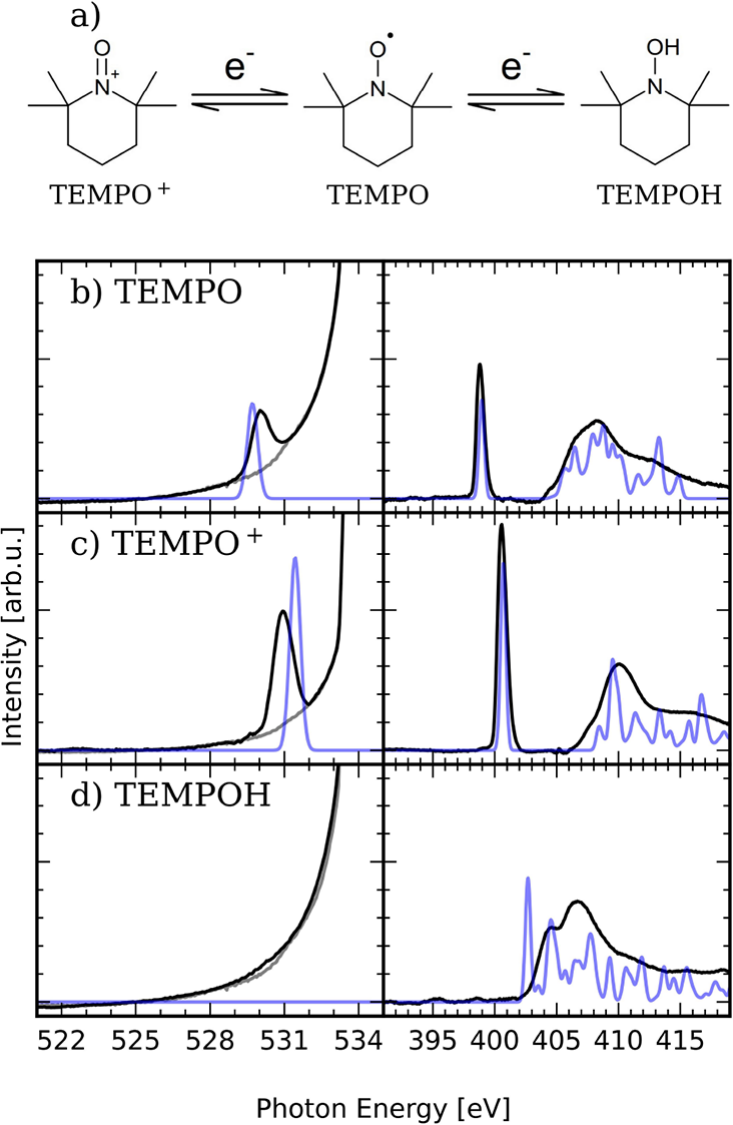
Electronic structure of TEMPO and its oxidized and reduced forms. (a) Structures of the redox forms of TEMPO­(center), TEMPO^+^ (left), and TEMPOH (right). The X-ray absorption spectra of (b) TEMPO, (c) TEMPO^+^, and (d) TEMPOH at the oxygen K-edge with a spectrum of water as a baseline in gray (left panels b–d) and nitrogen K-edge (right panels b–d), respectively. Experiment (black) and PBE0/TD-DFT calculations (blue). The X-ray absorption spectra were taken using 70 mM solutions of TEMPO, TEMPO^+^, and TEMPOH in water.

To determine the quenching mechanism, an aqueous solution of TEMPO was pumped with a laser wavelength of 257 nm to directly generate the radicals in situ. Using a shorter wavelength laser, water can be excited directly and ionized by a two-photon process. This simplifies the experiment as it allows us to have only water and the radical scavenger TEMPO present in the solution and thereby avoid potential side reactions between TEMPO and NO_2_^–^ and their photoproducts. These reactions could interfere with the scavenging process or overlap with the signals and thereby obscure the scavenging process. In contrast to Figure 2b, it can be seen in Figure 4b that OH^·^ is formed directly in water as a result of the laser irradiation without the addition of other reactants. However, TEMPO also absorbs at 257 nm. This causes two side reactions. First, TEMPO is excited into the second excited state and immediately decays into the lowest doublet excited state D_1_ on subpicosecond time scales. This lowest excited state (TEMPO*) can be seen on the oxygen K-edge (526.95 eV, green line) with a very short lifetime below the time resolution of our experiment of 80 ps (green trace in Figure 4g), before decaying back into the ground state (red trace in Figure 4g), where a short-lived intensity decrease is found on the early time scales, caused by the relaxation of the excited state back into the electronic ground state. Second, the excited state dissociates into atomic oxygen and the 2,2,6,6-tetramethylpiridine radical (TEMP). The TEMP radical can be observed at the nitrogen K-edge at 395.2 eV in agreement with the calculations (Figure 4f). The signals of OH^·^ (526 eV, magenta) and at 527.3 eV (purple) in Figure 4g, which lie right beside the excited state, show small intensity increases on the short time scales, which are the result of the overlap between the excited state signal and the neighboring peaks. At 527.3 eV (purple line), a short-lived state with a lifetime of τ = 1.9 ± 0.2 ns can be seen right after the laser excitation at 0.05 ns delay. The O 1s → 2p transition of atomic oxygen has previously been found at 527.3 eV in irradiated ice^38^ and at 526.8 eV in the gas phase.^39^ It has also been shown that TEMPO can be degraded to TEMP during the charge–discharge cycles of a redox flow battery.^40^ It is assumed that the oxygen rapidly reacts with the surrounding water to form other reactive oxygen species like OH^·^. Therefore, these signals are attributed to the dissociation products of TEMPO into TEMP and atomic oxygen.

**Figure 4:**
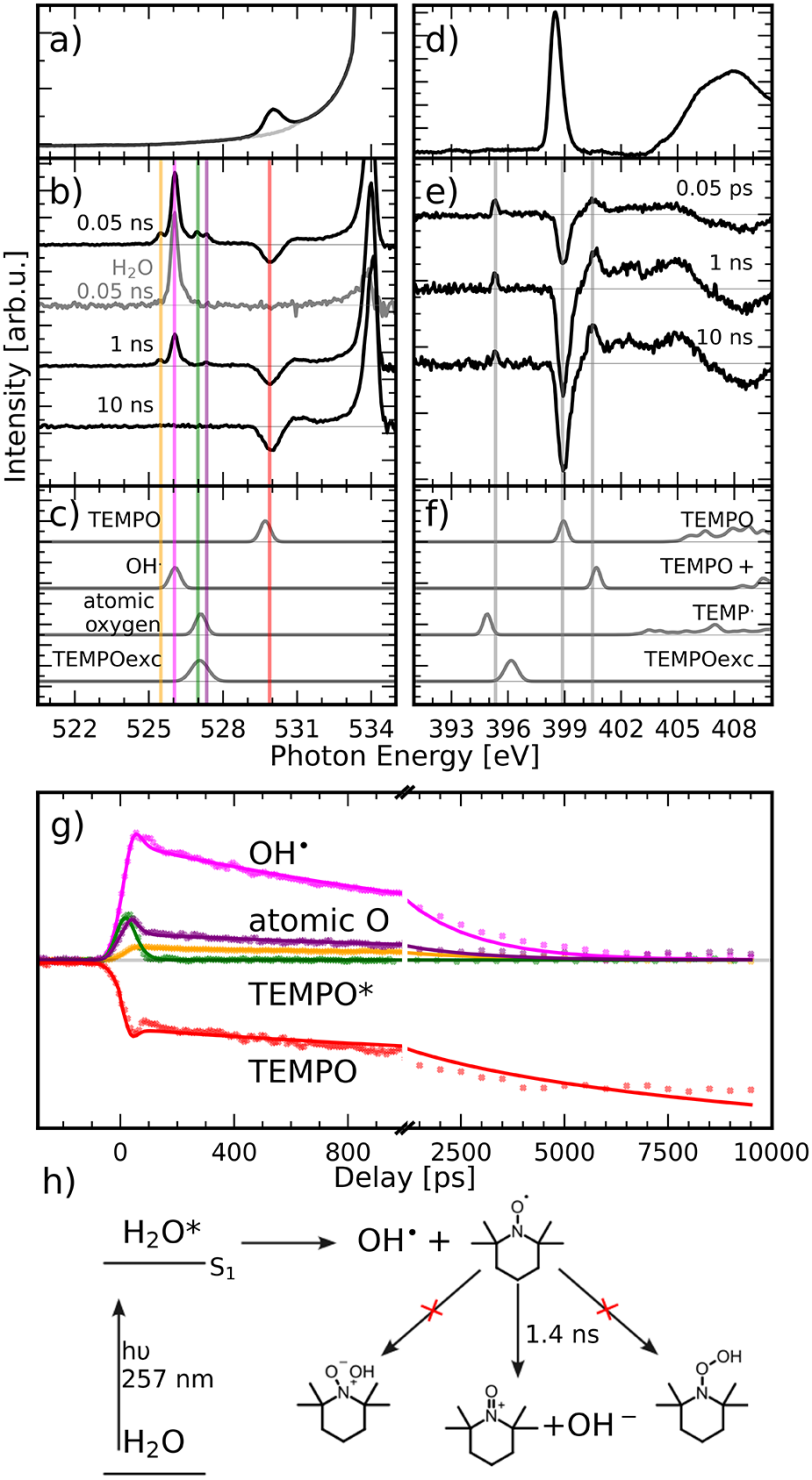
Dynamic evolution of TEMPO and its redox forms in aqueous solution from time-resolved X-ray absorption after photoexcitation at 257 nm. (a) Static oxygen K-edge spectrum of the ground state showing a π* absorption at 531.65 eV. (b) Oxygen K-edge pump probe spectra of TEMPO and H_2_O taken at different delays, energies at 525.45, 526, 526.95, 527.3, and 529.8 eV marked by yellow, magenta, green, violet, and red lines, respectively. (c) Calculations of O 1s → HOMO transition for different species using PBE0/TD-DFT. (d) Static nitrogen K-edge spectrum of the ground state showing a π* absorption at 398.6 eV. (e) Nitrogen K-edge pump probe spectra of TEMPO taken at different delays, with energies at 395.05, 398.6, and 400.2 eV, marked by vertical lines. (f) Calculations of N 1s → HOMO transition for different species using PBE0/TD-DFT. Spectra are offset vertically for visibility. (g) Time traces from 0.2 ns before excitation at 257 nm until 10 ns after excitation. The selected energies correspond to the energies marked in (b) and (c) by vertical lines; for details, see Discussion. All traces are assigned to species. Orange indicates a non-negligible screening interaction between OH^·^ and the nitroxyl group of TEMPO. (h) Summary of the mechanisms involved in OH^·^ scavenging by TEMPO. The static and transient X-ray absorption spectra were taken using 70 mM TEMPO in water.

The main bleach on both oxygen and nitrogen K-edges can be assigned to TEMPO, from both the dissociation reaction and the radical scavenging reaction with OH^·^. In the scavenging reaction, the two radicals TEMPO and OH^·^ form TEMPO^+^ and OH^–^ through electron transfer. The literature indicates that OH^–^ should show a feature at 532.5 eV.^41,42^ However, no such feature appears to be present in Figure 4b. This might be explained by the concentration of the sample and the intensity of the signal reported in the literature. Cappa et al.^41^ conducted their measurements with a 6 M solution of OH^–^ and were only able to detect a weak signal of OH^–^, while Chen et al.,^42^ could not detect OH^–^ below a concentration of 1 M. Considering that the concentration of OH^–^ is expected to be many times lower than those measured in the literature, it can be assumed that the concentration is too low to be detectable in our experiment. The signal of OH^·^ at 526 eV is present after excitation of water using the 257 nm laser in both the TEMPO-containing sample and pure water as a reference, unlike after excitation at 343 nm, where no OH^·^ was formed in pure water. The lifetime of OH^·^ in the presence of TEMPO is τ = 1.7 ± 0.2 ns, whereas the lifetime of OH^·^ in water without any radical scavengers (Figure 2g) is τ = 11.2 ± 0.5 ns.^12^ These lifetimes are in good agreement with the ones obtained from the aforementioned measurements using H_2_O_2_ and MeOH (Figures S1 and S2) and indicate that the scavenging of OH^·^ by TEMPO happens at or near diffusion-controlled rates. As a product of the reaction of TEMPO with OH^·^, TEMPO^+^ is formed, visible at 401.2 eV on the nitrogen K-edge and at 531 eV on the oxygen K-edge (Figure 4 b,e).

For the scavenging mechanism, two pathways have been proposed in the literature with the bonding of the OH^·^ to either the oxygen or the nitrogen of the nitroxyl radical, as is shown in Figure 4h.^12,43^ Arguments in favor of an oxygen-centered scavenging pathway are the sterically bulky methyl groups shielding the nitrogen from possible attack, which is one of the factors contributing to the stability of nitroxyl radicals both in solution and bulk material at room temperature. Also, while a product of TEMPO and OH^·^ is too unstable to be isolated, reaction products of nitroxyl radicals with carbon-centered radicals are stable at room temperature and can be analyzed by various spectroscopic techniques. These methods show that for carbon-centered radicals, the attack happens exclusively at the oxygen.^44–46^ However, oxygen is far more electronegative than carbon, and therefore, from an electronic point of view, an attack by an oxygen-centered radical appears far more likely on the electron-rich and more electropositive nitrogen than on the oxygen.

Another feature is found below OH^·^ in Figure 4b at 525.5 eV (orange line) with a lifetime τ = 2.2 ± 0.2 ns. This is unexpected, as the only oxygen-containing species in water with a lower core excitation energy than OH^·^ is H_2_O^+^.^47^ However, because H_2_O^+^ has a lifetime of 46 fs, this signal has to belong to a different species.^47^ Further, our calculations indicate that neither an oxygen- nor nitrogen-bound intermediate shows signatures below 529 eV. We propose a non-negligible screening interaction between OH^·^ and the nitroxyl group of TEMPO. This interaction would cause a small shift in electron density within the radical toward the oxygen, slightly lowering the core excitation energy. Calculations of the two radicals in proximity indeed show a weak coordination of the hydrogen in OH^·^ toward the oxygen in TEMPO and a resulting shift of approximately 0.3 eV in the oxygen K-edge absorption spectrum relative to solvated OH^·^ (Figure S1). The expected corresponding shift in the core excitation energy of TEMPO might not be visible due to the relative broadness of the bleach compared to the OH^·^ signal. This assignment would indicate that no long-lived bound intermediate between TEMPO and OH^·^ is formed, in contrast to the scavenging of other radicals like peroxyl radicals,^48^ carbon-centered radicals,^49^ and radicals of biomolecules.^45^ Instead, the scavenging of OH^·^ does not appear to occur through any intermediate structure as has been proposed before, but rather by electron transfer in a weakly coordinated state.

## Conclusion

In this work, we have investigated the mechanisms of the photoinduced generation of OH^·^ from NO_2_^–^ and the scavenging of OH^·^ by TEMPO using time-resolved soft X-ray absorption spectroscopy on the nitrogen and oxygen K-edges in water using a liquid flat-jet system. We have shown that the mechanism of the photodissociation of NO_2_^–^ results in the formation of NO^·^ and OH^·^ and determined the lifetime of the generated OH^·^ to be τ = 11.2 ± 0.5 ns in the absence of any radical scavengers, and compared it to the lifetime of OH^·^ in the presence of MeOH at τ = 1.3 ± 0.1 ns. We have investigated the fate of OH^·^ after its photoinduced generation and did not find indications of a reaction between OH^·^ and NO_2_^–^ at the investigated concentrations, showing that the generation of NO_2_^·^ has to occur through a different pathway. We also used transient soft X-ray absorption spectroscopy to investigate the mechanism of scavenging of OH^·^ by TEMPO. Here, we found that TEMPO efficiently scavenges OH^·^ to form TEMPO^+^ and OH^–^. We determined the lifetime of OH^·^ to be τ = 1.7 ± 0.2 ns in the presence of 70 mM TEMPO, showing that the scavenging is limited by the diffusion of OH^·^. We could not detect any intermediate structure of OH^·^ bound to either nitrogen or oxygen of the nitroxyl group in TEMPO, but instead found that OH^·^ might be weakly coordinated to TEMPO, resulting in a 0.5 eV shift in the core excitation of OH^·^ from 526 to 525.5 eV, showing that the mechanism does not occur through an intermediate, in which OH^·^ is bound to either the oxygen or nitrogen of the nitroxyl group and is instead an electron transfer between the two molecules without the formation of a chemical bond.

## Supplementary Material



## Notes

Additional transient X-ray absorption spectra, experimental and computational details, materials, and methods (PDF)
