# Supplementary material for: Mechanisms of Hydroxyl Radical Chemistry in Aqueous Solution Triggered by Photoexcitation and Probed by Soft X‑rays
Source: J Am Chem Soc. 2026 Feb 17;148(8):8567–73. doi: 10.1021/jacs.5c20053 (PMC12964397; doi:10.1021/jacs.5c20053)
Supplement: Supplementary file 1 [file ja5c20053_si_001.pdf]

Mechanisms of Hydroxyl Radical Chemistry in Aqueous Solution Triggered by Photoexcitation and Probed by Soft X-rays

Leo Cordsmeier,[*,†,‡] Wagner Ribeiro da Silva Neto,[‡] Mattis Fondell,[‡] Rolf Mitzner,[‡] Vinícius Vaz da Cruz,[‡] Sebastian Eckert,[*,‡] and Alexander Föhlisch[*,†,‡]

†*Universität Potsdam, Institut für Physik und Astronomie, Karl-Liebknecht-Straße 24/25, 14476 Potsdam, Germany*

‡*Helmholtz-Zentrum Berlin für Materialien und Energie GmbH, Institute for Methods and Instrumentation for Synchrotron Radiation Research, Hahn-Meitner-Platz 1, 14109 Berlin, Germany*

E-mail: leo.cordsmeier@helmholtz-berlin.de; sebastian.eckert@helmholtz-berlin.de; alexander.foehlisch@helmholtz-berlin.de

Experimental Details

All spectra were taken at BESSY II using the AXSYS-NEXAFS endstation at UE52-SGM[1] with the flatjet system.[2] For the nitrite measurements 500 mM of sodium nitrite (Santa Cruz Biotechnology, USA) was dissolved in deionized water. For the measurements of TEMPO (Sigma Aldrich, Germany) and its oxidized and reduced forms 70 mM of TEMPO, 2,2,6,6-Tetramethylpiperidine-1-oxoammonium nitrate and 1-Hydroxy-2,2,6,6-tetramethylpiperidine were dissolved in deionized water respectively. For the pump probe experiments a laser with

[1]Research carried out at BESSY II, Albert-Einstein-Straße 15, 12489, Berlin

a repetition rate of 208 kHz, a pulse length of approximately 300 fs, a spot size of $(75\times75)\mu m^2$ FWHM and a fundamental wavelength of 1030 nm was used in the third harmonic (343 nm) for nitrite and in the fourth harmonic (257 nm) for TEMPO. The pulse energies used were 3.9 μJ at both excitation wavelengths 257 nm and 343 nm. The spectra were calibrated in energy using the signature of N_2 dissolved in the water at 400.8 eV at the Nitrogen K-edge and the 4a1 peak of water at 535 eV at the Oxygen K-edge. The static X-ray absorption spectra were treated by first normalizing them by their thickness and then subtracting a water spectrum, except for $TEMPO^+$ where a spectrum of ${NO_3}^-$ in H_2O was used instead, and a calculated background accounting for sample except for their Nitrogen content.[3] UV-Vis spectra were taken using a Cary 5000 UV-Vis-NIR Spectrophotometer with a sample concentration of 0.5 mM $NaNO_2$ using quartz cuvettes with a sample thickness of 1 cm.

Curve Fitting Details

All time traces were fitted using a function consisting of a exponentially modified gaussian, as shown in Eq.1, after the subtraction of a background that is caused by a drift of the detector on longer timescales above 10 ns.

$$f(x, A, \mu, \sigma, \tau) = \frac{A}{\tau} \cdot exp\left(\frac{(x-\mu)-\frac{\sigma^2}{\tau}}{\sigma}\right) \cdot erfc\left(\frac{\mu}{\tau} + \frac{\sigma^2}{2\tau^2} - \frac{x}{\tau}\right) \tag{1}$$

In addition, the ground state bleach was fitted with a second exponentially modified gaussian with the same lifetime as the respective excited state to capture the internal conversion happening on short time scales. The lifetimes of the different species are given as the exponential time constant τ extracted from these fits with the fitting uncertainty given based on the standard error. In addition, a logarithmic growth function has been fitted to the time traces of $TEMPO^+$ in Figure S5 and NO in Figure S4 to better capture slow rise over longer time scales above 10 ns. To account for the overlapping of the excited state of TEMPO (green trace in Figure 4g) with the surrounding peaks on short time scales a second

exponentially modified gaussian has been fitted to the time traces of $OH^\bullet$ and atomic Oxygen in Figure 4g with the same shape and lifetime as the excited state TEMPO. The static spectra have been scaled by arbitrary factors compared to the transient spectra to improve visibility.

Computational Details

All theoretical calculations were performed using ORCA 5.0.3.[4,5] The PBE0 functional was used with the def2-TZVP(-f)[6] basis set, the def2/J[7] auxiliary basis set and Becke-Johnson damping.[8,9] The X-ray absorption spectra were calculated using the core-valence separation, by restricting the donor space in the linear response TD-DFT calculation to include the desired 1s orbital only. The XAS were calculated using optimized geometries obtained for the ground state of each species, while the geometry of the S1 excited states were optimized with TD-DFT. For calculations of XAS from excited states the restricted subspace approximation[10] was used.[11] This approach was originally designed to calculated RIXS spectra. However, it can also be used to calculate the core level → LUMO transition of the lowest excited state. Using RSA the virtual space was restricted to 20 unoccupied orbitals, while the occupied space reduced to the N1s or O1s and the 9 highest occupied orbitals. Then 200 roots were computed, populating all excitations from the valence and core level orbitals into the restricted virtual subspace. From this the X-ray absorption spectrum of the lowest excited state could be obtained, by calculating the transition dipole moments using Multiwfn.[12] This approach does not yield the full X-ray absorption spectrum but is limited to single electron transitions with respect to the ground state. Therefore, the calculated spectra of the molecules in their excited states lack features corresponding to multi-electron excitations. However, because our analysis focuses on the features with the lowest excitation energies, corresponding to a single electron transition to the optically depopulated orbitals, the calculated signal can still be used to assign a feature to the lowest excited state. For

all calculations implicit solvation using CPCM was used. Compared to the experiment the calculated spectra were convoluted with a gaussian broadening of 0.5 eV and were shifted by 13.275 eV on the Oxygen K-edge and by 11 eV on the Nitrogen K-edge.

Chemicals and Materials

2,2,6,6-Tetramethylpiperidine-1-oxoammonium nitrate was prepared by dissolving TEMPO (12 g, 76.8 mmol) in 300 mL Et_2O and slowly adding 68 % HNO_3 (Carl Roth, Germany, 5.4 mL, 88 mmol, 1.15 eq.). The solution was stirred for several minutes at room temperature and the yellow precipitate was filtered off. To the remaining solution 14 % NaOCl solution (Sigma Aldrich, Germany, 7.9 mL, 0.385 mmol, 0.5 eq.) was added and the solution was again stirred at room temperature. The aqueous phase was washed with Et_2O (Sigma Aldrich, Germany) and then extracted with Acetonitrile (Sigma Aldrich, Germany). The solvent was removed under vacuum and the product obtained in the form of yellow crystal (total yield 13 g, 78 %). The product was used in the measurements immediately without further purification.

1-Hydroxy-2,2,6,6-tetramethylpiperidine was prepared by dissolving TEMPO (3.1 g, 20 mmol) in 100 mL H_2O, then L-Ascorbic acid (Sigma Aldrich, Germany, 2.2 g, 12.5 mmol) was added. The solution was stirred at room temperature until it was fully discolored. The aqueous phase was extracted with Et_2O and the solvent removed under vacuum to obtain the product as white crystals (1.8 g, 58 %). The product was used in the measurements immediately without further purification.

Calculations of potential intermediate structures of TEMPO quenching $OH^{\bullet}$

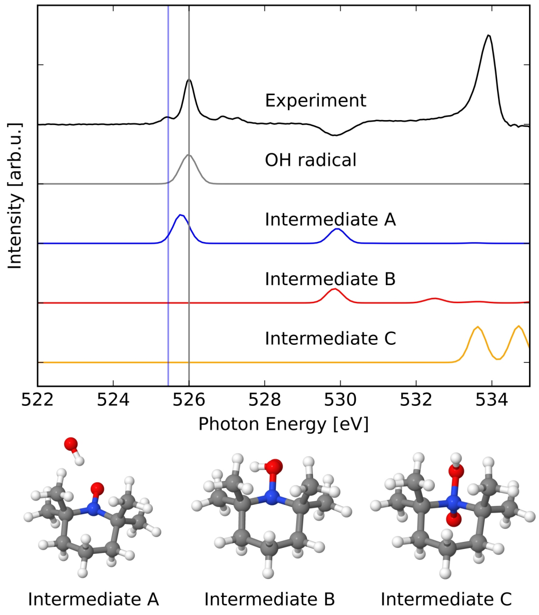

Figure S1: Oxygen K-edge pump probe spectra of TEMPO and H_2O taken at 50 ps delay after excitation and calculated X-ray absorption spectra for hydroxyl radical and three potential intermediary species in hydroxyl radical quenching by TEMPO, calculated using PBE0/TD-DFT.

The structure of three potential intermediates in the quenching on $OH^{\bullet}$ by TEMPO and the respective X-ray absorption spectra have been calculated and are shown in Fig. S1. Of these three the two structures B and C with $OH^{\bullet}$ bound to either Nitrogen or Oxygen of the nitroxyl group of TEMPO do not show any feature below 529 eV, where no features are found in the experimental data. In intermediate A, in which $OH^{\bullet}$ and TEMPO are only in close proximity the O1s $\rightarrow \pi^*$ transition is shifted by approximately 200 meV close to where another feature with a similar lifetime to $OH^{\bullet}$ is found at 525.5 eV in the experimental data. This might indicate that the quenching of $OH^{\bullet}$ might occur through a similar intermediate in which $OH^{\bullet}$ and TEMPO do not form a chemical bond.

X-ray absorption spectroscopy of H_2O_2

To determine the lifetime of $OH^\bullet$ in the presence and absence of any scavengers further experiments have been conducted using H_2O_2 and MeOH, the results of which are shown in Figure S2 and S3. In Figure S2a the static Oxygen K-edge absorption spectrum of H_2O_2 is shown with its UV-Vis spectrum in the inset. In Figure S2b transient X-ray absorption spectra for three different samples 70 ps after excitation at 257 nm are shown. All three spectra show a peak at 525.8 eV corresponding to $OH^\bullet$ being formed. In the samples containing H_2O_2 this peak is stronger and a bleach is visible at 532.2 eV, indicating that in these samples $OH^\bullet$ is not only formed from water, but also by excitation of H_2O_2. Further in the sample containing both H_2O_2 and MeOH another small peak can be seen at 530.3 eV, that is likely the scavenging product formed by the reaction of $OH^\bullet$ with MeOH. In the second experiment (Figure S3) the influence of varying concentrations of MeOH on the lifetime of $OH^\bullet$ is investigated. In the absence of any scavenger the lifetime of $OH^\bullet$ is $\tau = 10.1 \pm 0.3$ ns. Adding MeOH decreases the lifetime to $\tau = 6.2 \pm 0.1$ ns for 250 mM MeOH, $\tau = 2.5 \pm 0.1$ ns for 500 mM MeOH and $\tau = 1.3 \pm 0.1$ ns for 1 M of MeOH. Increasing the concentration of MeOH further has no more influence on the lifetime of $OH^\bullet$ indication that at this concentration the scavenging is entirely diffusion controlled. It can be noted that the decrease of the lifetime of $OH^\bullet$ with increasing concentrations of MeOH is approximately linear, indicating that the scavenging process of $OH^\bullet$ by MeOH is a single step process. From this experiment a maximum lifetime of $OH^\bullet$ of $\tau = 10.1 \pm 0.3$ ns in the absence of any scavenger and a minimum lifetime in the presence of an excess of MeOH of $\tau = 1.3 \pm 0.1$ ns can be extracted.

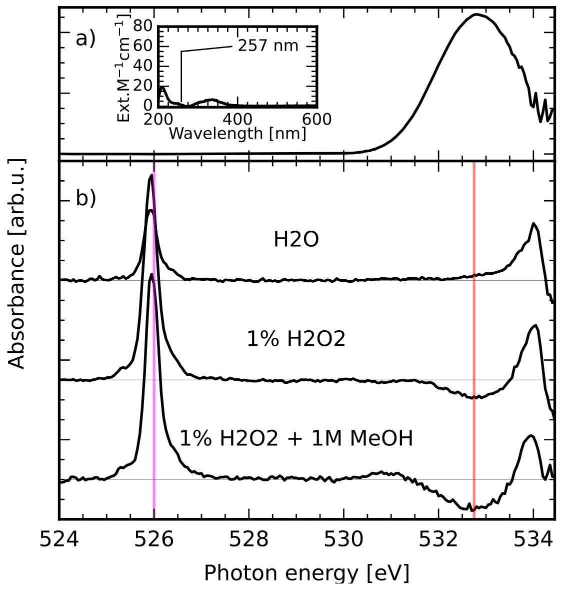

Figure S2: a) static Oxygen K-edge spectrum of the ground state of H_2O_2 showing absorption at 532.2 eV, the inset shows the UV-Vis spectrum of H_2O_2 b) Oxygen K-edge pump probe spectra of H_2O_2 and H_2O taken 70 ps after excitation at 257 nm, energies at 525.8 eV and 532.2 eV marked by magenta and red lines respectively.

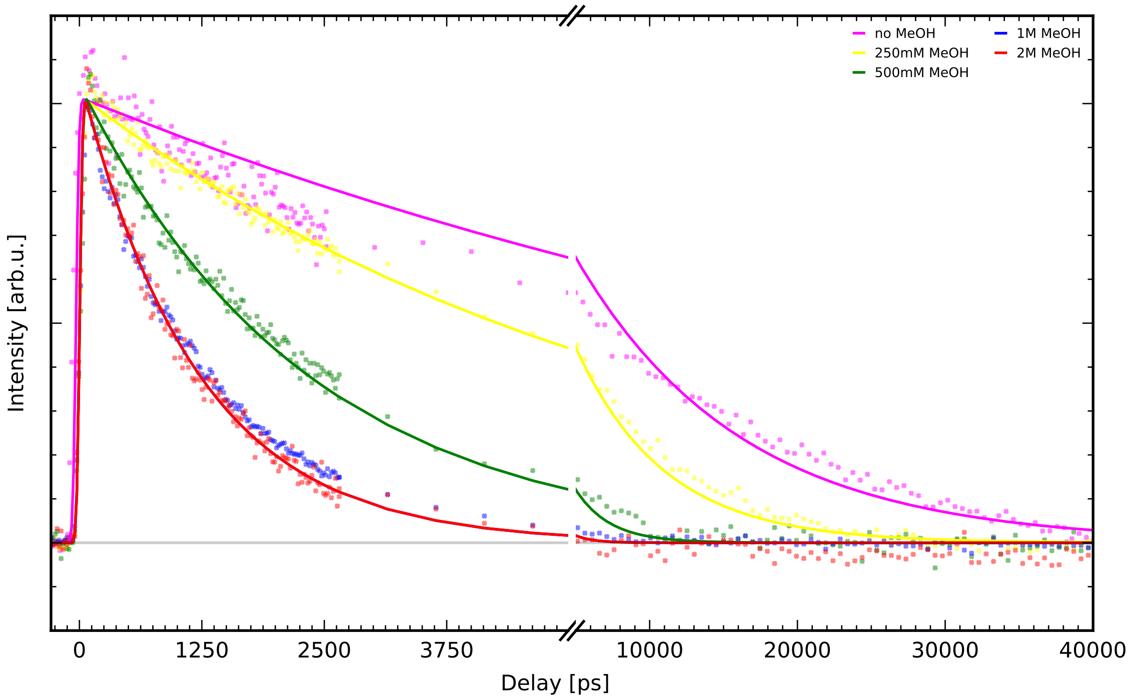

Figure S3: Time traces at 525.8 eV of an aqueous solution of H_2O_2 with different concentrations of MeOH.

Nitrogen K-edge time traces of $NO_2{}^-$

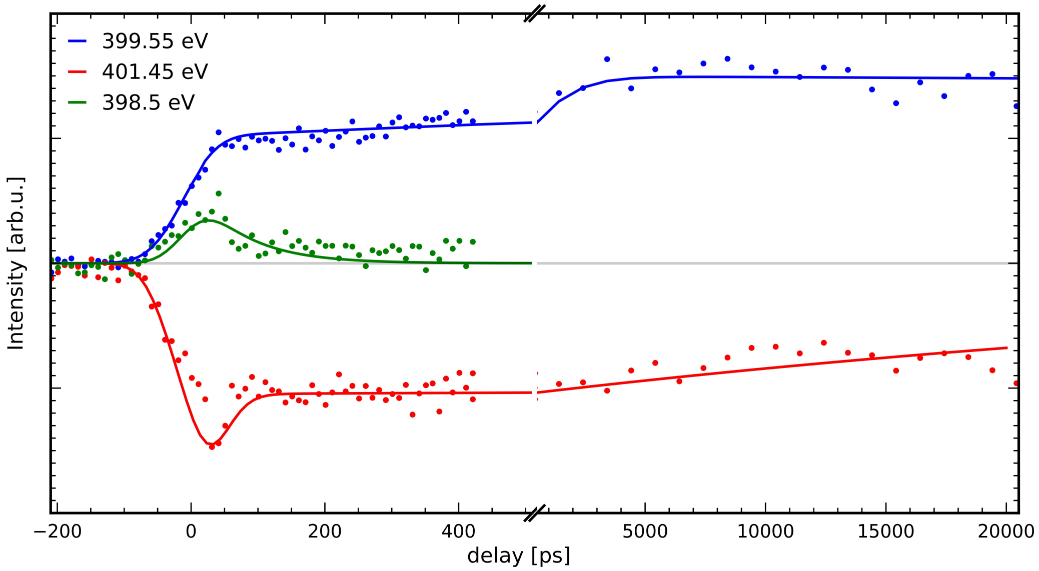

Figure S4: Time traces from 0.2 ns before excitation at 343 nm until 20 ns after excitation are shown. The selected energies correspond to the energies marked Figure 2e by vertical lines, the red trace corresponds to the ground state of $NO_2{}^-$, the blue trace corresponds to $NO^\bullet$ and the green trace to the first excited state of $NO_2{}^-$.

Nitrogen K-edge time traces of TEMPO

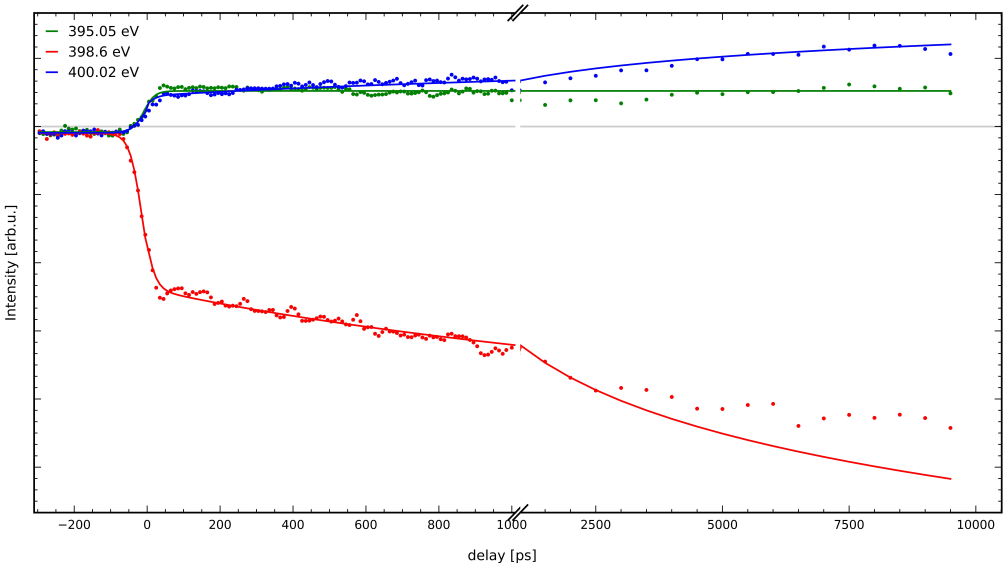

Figure S5: Time traces from 0.2 ns before excitation at 257 nm until 10 ns after excitation are shown. The selected energies correspond to the energies marked in Figure 4e by vertical lines, the red trace corresponds to the ground state of TEMPO, the blue trace to $TEMPO^+$ and the green trace to the TEMP radical.

References

(1) Miedema, P. S.; Quevedo, W.; Fondell, M. The variable polarization undulator beamline UE52 SGM at BESSY II. *Journal of large-scale research facilities JLSRF* **2016**, *2*, A70–A70.

(2) Fondell, M. et al. Time-resolved soft X-ray absorption spectroscopy in transmission mode on liquids at MHz repetition rates. *Structural Dynamics* **2017**, *4*, 054902.

(3) Henke, B.; Gullikson, E.; Davis, J. X-ray interactions: photoabsorption, scattering, transmission, and reflection at E=50-30000 eV, Z=1-92,. *At. Data Nucl. Data Tables* **1993**, *54*, 181–342.

(4) Neese, F. The ORCA program system. *WIREs Computational Molecular Science* **2012**, *2*, 73–78, _eprint: https://onlinelibrary.wiley.com/doi/pdf/10.1002/wcms.81.

(5) Neese, F. Software update: The ORCA program system—Version 5.0. *WIREs Computational Molecular Science* **2022**, *12*, e1606, _eprint: https://onlinelibrary.wiley.com/doi/pdf/10.1002/wcms.1606.

(6) Weigend, F.; Ahlrichs, R. Balanced basis sets of split valence, triple zeta valence and quadruple zeta valence quality for H to Rn: Design and assessment of accuracy. *Physical Chemistry Chemical Physics* **2005**, *7*, 3297–3305, Publisher: Royal Society of Chemistry.

(7) Weigend, F. Accurate Coulomb-fitting basis sets for H to Rn. *Physical Chemistry Chemical Physics* **2006**, *8*, 1057–1065, Publisher: Royal Society of Chemistry.

(8) Grimme, S.; Antony, J.; Ehrlich, S.; Krieg, H. A consistent and accurate ab initio parametrization of density functional dispersion correction (DFT-D) for the 94 elements H-Pu. *The Journal of Chemical Physics* **2010**, *132*, 154104.

(9) Grimme, S.; Ehrlich, S.; Goerigk, L. Effect of the damping function in dispersion corrected density functional theory. *Journal of Computational Chemistry* **2011**, *32*, 1456–1465, _eprint: https://onlinelibrary.wiley.com/doi/pdf/10.1002/jcc.21759.

(10) Cruz, V. V. d.; Eckert, S.; Föhlisch, A. TD-DFT simulations of K-edge resonant inelastic X-ray scattering within the restricted subspace approximation. *Physical Chemistry Chemical Physics* **2021**, *23*, 1835–1848, Publisher: Royal Society of Chemistry.

(11) Büchner, R.; Vaz Da Cruz, V.; Grover, N.; Charisiadis, A.; Fondell, M.; Haverkamp, R.; Senge, M. O.; Föhlisch, A. Fundamental electronic changes upon intersystem crossing in large aromatic photosensitizers: free base 5,10,15,20-tetrakis(4-carboxylatophenyl)porphyrin. *Phys. Chem. Chem. Phys.* **2022**, *24*, 7505–7511.

(12) Lu, T.; Chen, F. Multiwfn: A multifunctional wavefunction analyzer. *Journal of Computational Chemistry* **2012**, *33*, 580–592, _eprint: https://onlinelibrary.wiley.com/doi/pdf/10.1002/jcc.22885.
